# Appointment of L. J. Robert, M.D., to the Chair of Physiology and Pathology of the Oglethorpe Medical College at Savannah, Georgia

**Published:** 1856-09

**Authors:** 


☞ We perceive from the Circular of the Oglethorpe Medical College at Savannah, that L. J. Robert, M. D., of Marietta, Ga., has been appointed to the Chair of Physiology and Pathology, in that Institution.

